# Adherence to a Multidisciplinary Lifestyle Program for Patients With Atrial Fibrillation and Obesity: Feasibility Study

**DOI:** 10.2196/32625

**Published:** 2022-04-29

**Authors:** Nicole Tenbult, Jos Kraal, Rutger Brouwers, Ruud Spee, Sabine Eijsbouts, Hareld Kemps

**Affiliations:** 1 Telemedicine and Rehabilitation in Chronic Disease Flow, Center for Prevention Máxima MC Veldhoven/Eindhoven Netherlands; 2 Faculty Industrial Design Engineering Delft Netherlands; 3 Department of Cardiology Máxima MC Veldhoven/Eindhoven Netherlands; 4 Department of Industrial Design University of Technology Eindhoven Eindhoven Netherlands

**Keywords:** cardiac rehabilitation, atrial fibrillation, obesity, participation, completion, adherence, lifestyle

## Abstract

**Background:**

Atrial fibrillation is commonly associated with obesity. Observational studies have shown that weight loss is associated with improved prognosis and a decrease in atrial fibrillation frequency and severity. However, despite these benefits, nonadherence to lifestyle programs is common.

**Objective:**

In this study, we evaluated adherence to and feasibility of a multidisciplinary lifestyle program focusing on behavior change in patients with atrial fibrillation and obesity.

**Methods:**

Patients with atrial fibrillation and obesity participated in a 1-year goal-oriented cardiac rehabilitation program. After baseline assessment, the first 3 months included a cardiac rehabilitation intervention with 4 fixed modules: lifestyle counseling (with an advanced nurse practitioner), exercise training, dietary consultation, and psychosocial therapy; relaxation sessions were an additional optional treatment module. An advanced nurse practitioner monitored the personal lifestyle of each individual patient, with assessments and consultations at 3 months (ie, immediately after the intervention) and at the end of the year (ie, 9 months after the intervention). At each timepoint, level of physical activity, personal goals and progress, atrial fibrillation symptoms and frequency (Atrial Fibrillation Severity Scale), psychosocial stress (Generalized Anxiety Disorder–7), and depression (Patient Health Questionnaire–9) were assessed. The primary endpoints were adherence (defined as the number of visits attended as percentage of the number of planned visits) and completion rates of the cardiac rehabilitation intervention (defined as performing at least of 80% of the prescribed sessions). In addition, we performed an exploratory analysis of effects of the cardiac rehabilitation program on weight and atrial fibrillation symptom frequency and severity.

**Results:**

Patients with atrial fibrillation and obesity (male: n=8; female: n=2; age: mean 57.2 years, SD 9.0; baseline weight: mean 107.2 kg, SD 11.8; baseline BMI: mean 32.4 kg/m2, SD 3.5) were recruited. Of the 10 participants, 8 participants completed the 3-month cardiac rehabilitation intervention, and 2 participants did not complete the cardiac rehabilitation intervention (both because of personal issues). Adherence to the fixed treatment modules was 95% (mean 3.8 sessions attended out of mean 4 planned) for lifestyle counseling, 86% (mean 15.2 sessions attended out of mean 17.6 planned) for physiotherapy sessions, 88% (mean 3.7 sessions attended out of mean 4.1 planned) for dietician consultations, and 60% (mean 0.6 sessions attended out of mean 1.0 planned) for psychosocial therapy; 70% of participants (7/10) were referred to the optional relaxation sessions, for which adherence was 86% (mean 2 sessions attended out of mean 2.4 planned). The frequency of atrial fibrillation symptoms was reduced immediately after the intervention (before: mean 35.6, SD 3.8; after: mean 31.2, SD 3.3), and this was sustained at 12 months (mean 24.8, SD 3.2). The severity of atrial fibrillation complaints immediately after the intervention (mean 20.0, SD 3.7) and at 12 months (mean 9.3, SD 3.6) were comparable to that at baseline (mean 16.6, SD 3.3).

**Conclusions:**

A 1-year multidisciplinary lifestyle program for obese patients with atrial fibrillation was found to be feasible, with high adherence and completion rates. Exploratory analysis revealed a sustained reduction in atrial fibrillation symptoms; however, these results remain to be confirmed in large-scale studies.

## Introduction

Worldwide, atrial fibrillation is the most common sustained cardiac arrhythmia in adults [1]. It has been estimated that 17.9 million people will suffer this condition in Europe by 2060 [2]. The prevalence of atrial fibrillation also increases with the presence of traditional cardiovascular disease risk factors including diabetes, obesity, and hypertension [3]. Importantly, several longitudinal studies [4,5] have demonstrated the association between obesity and cardiac dysrhythmias with increased risk of atrial fibrillation. Moreover, patients with obesity often have a higher burden and faster progression of atrial fibrillation symptoms compared with patients having a normal weight. Not surprisingly, sustainable weight loss is, therefore, associated with a decrease in atrial fibrillation symptoms [6]. Lifestyle modification, including education, psychosocial support, dietary counseling, and physical training, are effective strategies to achieve weight loss in order to reduce atrial fibrillation symptoms and improve quality of life [7]. European Society of Cardiology guidelines [8] for the management of atrial fibrillation recommend lifestyle modification to achieve weight reduction for obese patients with atrial fibrillation; however, in practice, the effects of traditional lifestyle modification programs are often not maintained in the long-term because regular cardiac rehabilitation programs, which focus on restoring the physical condition, improving lifestyle, and providing psychological support throughout, do not sufficiently focus on behavior change [9]. In addition, only a small proportion of patients with atrial fibrillation are included in cardiac rehabilitation programs, despite proven clinical benefits [10,11]. Low participation rates are caused by suboptimal referral by medical professionals. In addition, participation in cardiac rehabilitation programs for patients with atrial fibrillation is not reimbursed by health insurance in the Netherlands. Although at the start of cardiac rehabilitation, more than 80% of patients are overweight and more than 50% have metabolic syndrome [12], most cardiac rehabilitation programs do not contain a program specifically aimed at weight loss [12,13]. In addition, most efforts to change health behaviors have had limited success due to dropout and declines in adherence [14]. Therefore, addressing barriers that influence adherence, such as a lack of personalization or tailoring of the program to a specific disease is key for improving the effectiveness of lifestyle modification programs [15,16].

In this study, we aimed to investigate the adherence and feasibility of a lifestyle program for overweight atrial fibrillation patients specifically designed to improve long-term adherence by focusing on improving self-management and sustainable behavior change.

## Methods

### Study Design

We conducted a feasibility study; the results of this study will be used to optimize the intervention, specifically targeted to long-term behavior change, in an associated parallel multicenter open-label randomized controlled trial (Optimal Cardiac Rehabilitation XL; Netherlands Trial Register NL5589) that will evaluate the cost-effectiveness of a multidisciplinary cardiac rehabilitation program in obese patients with atrial fibrillation and coronary artery disease.

### Ethics

The study protocol was reviewed and approved by the medical research ethics committee of Máxima Medical Center. All participants were informed about the goals of the study, and all participants provided written informed consent.

### Participants

Patients with atrial fibrillation who presented at the cardiac rehabilitation unit (Centre Flow, Máxima Medical Center in Veldhoven/Eindhoven) in 2016 and 2017 were eligible for participation. Patients with atrial fibrillation and obesity were referred by the cardiologist, and subsequently, invited for an intake assessment with an advanced nurse practitioner. All participants were screened for trainability and practical possibilities. To personalize the lifestyle intervention, participants were counseled to formulate personal lifestyle goals based on SMART (Specific, Measurable, Achievable, Realistic and relevant, and Timed [17]), for a 1-year lifestyle program consisting of physical training, nutrition guidance, lifestyle counseling, and psychosocial therapy, with relaxation therapy as optional additional treatment module.

For this study, we chose a sample of 10 participants, rather than using a calculation-based sample size because the goal of this study was not to evaluate clinical effectiveness but to evaluate the feasibility of the intervention in this specific patient population.

### Inclusion and Exclusion Criteria

Patients with symptomatic atrial fibrillation and a BMI higher or equal to 29 kg/m^2^ were asked to participate in the study. Exclusion criteria were progressive symptoms of heart failure, myocardial ischemia during exercise testing (ST depression ≥2 mm), sinus tachycardia at rest with a frequency greater than 110 bpm or atrial fibrillation greater than 100 bpm at rest, and severe cognitive impairment (memory, attention, concentration and psychiatric disorders).

### Intervention

An individual program was created with the patient, based on their exercise capacity and personal goals. To improve exercise capacity, aerobic training was included, as well as strength training. Training frequency, training duration, training intensity, length of the work and rest intervals, and dosage training were used as variables to determine prescribed aerobic and strength training. The first 6 weeks of the intervention consisted of 2 training sessions per week, and the 6 weeks thereafter consisted of training sessions once per week. Blood pressure and heart rate were checked before, during, and after sessions. Training sessions (total duration: 60 minutes) consisted of aerobic training (treadmill or bicycle ergometer; a build-up time of 2 weeks at 40% to 50% of maximum rate of oxygen consumption was recommended) and strength training. For each strength training exercise (seated leg press, chest press, biceps curls, triceps curls, core muscle exercises, low-dose hip abductor-adductor exercise, and low row back exercise), 2 sets were performed with 1 to 2 minute breaks in-between, and each set consisted of 12 repetitions. In addition to the exercise program, all participants received an individual intake assessment with a dietician and a psychologist. If indicated (ie, depending on care need), treatments were planned with the dietician or psychologist. Finally, participants were offered an optional relaxation module, which consisted of breathing exercise and body awareness relaxation techniques aimed at teaching participants to create more moments for rest and to cope better with stress.

At the end of the 3-month intervention and at the end of the year, participant progress was evaluated with the advanced nurse practitioner, and personal goals were adjusted (participants were coached on active lifestyle and personal goals). Participants were instructed to continue to pursue these goals in their home environment. We used behavior change techniques such as goal setting, action planning, motivational interviewing, information about healthy lifestyle and reviewing outcome goals.

### Main Outcomes and Measures

The primary endpoints of the study were percentage of participants who completed the intervention and adherence to the treatment modules (percentage of visits attended out of those planned). Secondary endpoints included body weight, atrial fibrillation frequency and severity, exercise capacity, anxiety, and depression. The frequency and symptom severity of atrial fibrillation were assessed with the Atrial Fibrillation Severity Scale, which is a validated questionnaire [18] that combines measures of frequency and patient-perceived severity. Exercise capacity was measured using the 6-minute walking test. The 6-minute walking test was performed in a 25-meter-long corridor at a speed of the participants’ preference with the instruction to cover the greatest possible distance during 6 minutes without running. The 1-repetition maximum, a measure of muscle strength, was defined as the maximum weight that could be pushed or lifted once. Anxiety and depression complaints were assessed using validated questionnaires (Patient Health Questionnaire–9 [19] and Generalized Anxiety Disorder–7 [20]). Weight and height were measured at all follow-up visits, and BMI was calculated. Secondary outcome measures were assessed at baseline, at 3 months (ie, immediately after the intervention), and at 12 months.

### Statistical Analysis

Program completion and adherence to the treatment modules and changes in secondary endpoints (body weight and Atrial Fibrillation Severity Scale score) were analyzed by calculating descriptive statistics.

## Results

A total of 10 participants participated in the feasibility study. The majority of participants were male (male: n=8; female: n=2; age: mean 57.2 years, SD 9.0; baseline weight: mean 107.2 kg, SD 11.8; baseline BMI: mean 32.4 kg/m^2^, SD 3.5). Two participants did not complete their cardiac rehabilitation programs due to personal problems.

Of the 10 participants, 8 participants completed all 4 appointments with the advanced nurse practitioner to monitor their lifestyle (mean 3.8, SD 0.42 appointments attended per participant); 2 participants canceled their last appointment, resulting in an adherence of 95% (mean 3.8 sessions attended out of mean 4 planned). All participants were referred to the exercise training program sessions, for which adherence was 86% (mean 15.2 sessions attended out of mean 17.6 planned); 9 participants were referred to a dietician (1 patient had already received dietetic treatment in a referring hospital), for which adherence was 88% (mean 3.7 sessions attended out of mean 4.1 planned); and all participants were referred to a medical psychologist, for which adherence was 60% (mean 0.6 sessions attended out of mean 1.0 planned). Of the 10 participants, 9 participants were referred to, of whom 7 participants participated in, relaxation sessions for which adherence to the relaxation sessions was 86% (mean 2 sessions attended out of mean 2.4 planned).

After the initial 3-month cardiac rehabilitation period, weight decreased from mean 107.2 kg (SD 11.8) to mean 102.5 kg (SD 13.7), and there was weight loss at 12 months compared with baseline (Table 1). The frequency of atrial fibrillation symptoms was reduced immediately after the intervention (before: mean 35.6, SD 3.8; after: mean 31.2, SD 3.3), and this was sustained at 12 months (mean 24.8, SD 3.2). Severity of atrial fibrillation complaints immediately after the intervention (mean 20.0, SD 3.7) and at 12 months (mean 9.3, SD 3.6) were comparable with that at baseline (mean 16.6, SD 3.3). Depression and anxiety scores immediately after the intervention were comparable with those at baseline. The distance walked on the 6-minute walking test was higher after the intervention (before: mean 538.9, SD 70.6 meters; after: mean 595.3, SD 72.8 meters) and at 12 months (mean 600.8, SD 94.0). Quadriceps strength (1-repetition maximum) showed an increase immediately after rehabilitation (before: mean 179.5, SD 49.3 kg; after: mean 245.3, SD 63.7 kg) and at 12 months (mean 259.7, SD 52.7 kg). The 1-repetition maximums for biceps (before: mean 22.1, SD 8.9 kg; after: mean 25.6, SD 6.0 kg) and triceps (before: mean 27.1, SD 9.7 kg; after: mean 35.6, SD 6.8 kg) also showed increases in strength immediately after cardiac rehabilitation, and at 1 year, remained higher (biceps: mean 29.8, SD 3.4, triceps: mean 39.6, SD 2.5) than those at baseline.

**Table 1:** Outcome measures at baseline, at 3 months (immediately after the cardiac rehabilitation intervention), and at 12 months (follow-up).

			Baseline, mean (SD)		Postintervention (at 3 months), mean (SD)		Follow-up (at 12 months), mean (SD)
Weight (kg)			107.2 (11.8)		102.5 (13.7)		105.1 (16.1)
**Atrial Fibrillation Severity Scale**							
	Atrial fibrillation frequency	35.6 (3.8)		31.2 (3.3)		24.8 (3.2)	
	Atrial fibrillation severity	20.0 (3.7)		16.6 (3.3)		9.3 (3.6)	
	6-Minute walking test (m)	538.9 (70.6)		595.3 (72.8)		600.8 (94.0)	
	Patient Health Questionnaire–9	6.8 (4.3)		4.6 (4.3)		—^a^	
	Generalized Anxiety Disorder–7	4.4 (5.3)		2.7 (2.9)		—	
**1-Repetition maximum**							
	Quadriceps (kg)	179.5 (49.3)		245.3 (63.7)		259.7 (52.7)	
	Biceps (kg)	22.1 (8.9)		25.6 (6.0)		29.8 (3.4)	
	Triceps (kg)	27.1 (9.7)		35.6 (6.8)		39.6 (2.5)	

^a^No data.

## Discussion

### General

This study shows that a multidisciplinary lifestyle program focused on sustainable behavior change in individuals who are overweight with atrial fibrillation is feasible with high completion rates (8/10 participants, 80%) and high adherence to the treatment modules (60% to 95%). Although exploratory analysis revealed sustained improvements in both the frequency and the severity of the atrial fibrillation symptoms and modest weight reduction, these results need to be confirmed in large-scale randomized controlled trials.

To the best of our knowledge, this is the first study to evaluate cardiac rehabilitation adherence specifically in obese patients with symptomatic atrial fibrillation. One study [21] showed that 81.3% of general patients referred for cardiac rehabilitation attended at least one-half of the sessions; however, nearly half of the patients did not follow any dietary recommendations or did not increase their physical activity levels, and in another, the dropout rate (24% [22]) was comparable to that in our study (20%). However, whereas participation in exercise training was high (90% [22]), participation in other modules (25% for dietary counseling, 9% for relaxation therapy, 9% for psychological therapy [22]) was substantially lower than those in our study. Data on adherence to the planned sessions were lacking [22].

The relatively high completion and adherence rates in our study may be explained by the design of the intervention. First, the intervention was designed to be a nurse-coordinated multidisciplinary intervention, rather than one that focused on exercise training or dietary counseling only, which is in line with the notion that holistic care models targeting are most effective for behavior change [23]. Second, counseling on expectations and individualized goal setting constituted an important part of the intervention. In fact, a previous study [24] showed that unrealistic expectations form an important barrier to lifestyle behavior change [24]. Also, it is well recognized that goal setting is an effective way of achieving behavior change [24,17]. Third, the high adherence rates may be explained by the fact that we used motivational interviewing, which is a communication skill set to help facilitate change and adherence to health behaviors. In fact, Pack et al [25] showed that continual, purposeful, and planned quality improvement efforts significantly increased patient participation in cardiac rehabilitation—motivational interviewing was an important strategy in achieving this.

Our exploratory analysis revealed that cardiac rehabilitation resulted in sustained reductions in the frequency and severity of atrial fibrillation symptoms. However, in contrast with other studies [6,26,27] that have shown that weight loss has beneficial effects on atrial fibrillation symptoms, the reduction in symptoms in our study was not associated with weight loss, suggesting that other physiological mechanisms, such as improvement of traditional cardiovascular risk factors and an increase in physical fitness, play a role. In line with this assumption, Pathak et al [6] observed a significant dose-response relationship between improvements in physical fitness and reduction in the risk of atrial fibrillation recurrence. Other studies [26,28] have shown positive effects of comprehensive management of concomitant cardiovascular risk factors on atrial fibrillation recurrence. Although these studies [26,28,29] underline that targeting modifiable risk factors, obesity and physical fitness should be an essential pillar of atrial fibrillation care, the optimal approach to achieve sustained long-term effects in obese patients with atrial fibrillation remains to be established.

### Limitations

An important limitation of this study was the small number of study participants. We, therefore, described conclusions only about feasibility and not about the outcomes. Furthermore, a control group was not included because the primary goal of this study was to evaluate feasibility and adherence; therefore, the reported effects of the intervention on weight and atrial fibrillation symptoms should be regarded as exploratory. The results of this feasibility study will applied to an ongoing study.

### Conclusions

This study showed that a nurse-coordinated multidisciplinary lifestyle program for obese patients with atrial fibrillation is feasible, with a high level of completion and adherence. Effects on atrial fibrillation symptoms and weight reduction remain to be determined in a randomized controlled trial.

## Abbreviations

BMI: body mass index
